# Bilateral Complete Loss and Partial Regeneration of Photoreceptor Layers in a Pediatric Vogt-Koyanagi-Harada (VKH) Case

**DOI:** 10.7759/cureus.32273

**Published:** 2022-12-06

**Authors:** Shaikha Aldossari, Nawaf A Alhussaini, Abdulrahman Barri, Hassan Al Dhibi

**Affiliations:** 1 Ophthalmology, King Khaled Eye Specialist Hospital, Riyadh, SAU; 2 College of Medicine, King Saud Bin Abdulaziz University for Health Sciences, Riyadh, SAU; 3 Internal Medicine, King Saud University College of Medicine, Riyadh, SAU

**Keywords:** vogt-koyanagi-harada disease, photoreceptors regeneration, loss of photoreceptors, pediatric, hyperglycemia with steroid, central diabetes insipidus

## Abstract

Vogt-Koyanagi-Harada (VKH) disease is a multisystem autoimmune disease affecting melanocyte-containing tissues in the eyes, meninges, ear, and skin. As far as we are aware, this is a unique case report documenting the regeneration of the photoreceptor layer after bilateral complete loss of the photoreceptor layer in a child with VKH. We report a case of a 12-year-old male with no significant past medical history who presented during the chronic stage of incomplete VKH. He was found to have a complete loss of photoreceptor layer in both eyes during a work-up to confirm the aforementioned disease. Upon receiving his first pulse dose of 500 mg IV methylprednisolone as a treatment course, he developed severe steroid-induced hyperglycemia (random blood glucose of 17.6 mmol/L). Additionally, a brain MRI revealed pituitary gland changes compatible with diabetes insipidus, which is a combination that was mentioned once in the literature before. A review of the systems did not suggest any other systemic diseases. The patient’s elevated blood sugar level was managed by a pediatrician, and it normalized. At his last visit, optical coherence tomography (OCT) showed hypertrophy/regeneration of the photoreceptor layer. This case report indicates that retinal photoreceptor layer regeneration can be sometimes observed with follow-up after the resolution of inflammation and uveitis.

## Introduction

Vogt-Koyanagi-Harada (VKH) disease is a multisystem autoimmune disease affecting melanocyte-containing tissues in the eyes, meninges, ear, and skin [1]. In the eyes, it specifically affects the choroid layer. It rarely affects children. It accounts for 22% of cases of uveitis attending our hospital (King Khaled Eye Specialist Hospital) between the years 2001 and 2010 [2]. At least three of the following four signs should be present to diagnose the disease; bilateral chronic iridocyclitis, posterior uveitis, neurological signs, and/or cutaneous signs [1]. The clinical course of the disease follows four stages, starting with a prodromal stage that mimics viral illnesses followed by an acute uveitic phase, then a convalescent phase which can proceed to a chronic recurring phase [1].

## Case presentation

A 12-year-old boy, a product of an uneventful full-term cesarean section with no history of developmental delays, was not on any medications and has no known allergies. His vaccinations were up to date with an unremarkable family history of autoimmune diseases. He presented to our emergency department with a gradual decrease in vision over one year with a two-week history of bilateral eye redness that was associated with headache and bilateral blurring of vision. His family also reported a history of on/off fever, cough, joint pain, lower abdominal pain, dark urine, headache, and palpitations lasting for one year.

A general examination that was done by a pediatrician around the time of the presentation revealed a patient who was in good condition having a low-grade fever of 37.8 degree celsius. Upon admission, ejection systolic murmur was heard along the left sternal border with otherwise normal ear nose throat, neck, chest cardiovascular, abdomen, joints, skin, and neurological examinations. Cyclorefraction was: right eye: -0.75-3.25 x180; left eye: -0.50-2.50 x005, interpupillary distance (IPD): 67 mm, and normal intraocular pressure (IOP) throughout his visits.

Ophthalmology exams throughout his follow-ups are summarized in Table 1.

**Table 1:** Ophthalmology exams throughout patient’s follow-up visits. BCVA, best corrected visual acuity; IOP, intraocular pressure; SLE, slit lamp examination; f/u, follow up; PH, pinhole; CF, count fingers vision; OD, right eye; OS, left eye; OU, both eyes; AC, anterior chamber; Conj, conjunctiva; RD, retinal detachment; ERD, exudative retinal detachment; RPE, retinal photoreceptor epithelium

Date	General	BCVA	IOP	SLE	Fundus
First visit (Jan 28^th^, 2018)	White patch of hair	(Dilated with PH) 20/80, 20/125	13 OU	Conjunctival injection, +2 cells in AC, +1 cells in vitreous	Hyperemic discs, exudative RD inferiorly, no breaks
Day 3 post-therapy (Feb 2^nd^, 2018)	Same	(Dilated with PH) 20/80, 20/125	8 OU	Conj: Quiet, Cornea: clear, AC: +1 cells/deep Broken posterior synechia OS	No vitritis, ERD mainly inferiorly
Day 4 post-therapy (Feb 3^rd ^, 2018)	Same	(Dilated with PH) 20/100, 20/125	12.5, 14.5	Conj: Quiet, Cornea: clear, AC: +2 cells/deep Broken posterior synechia OS	No vitritis, ERD mainly inferiorly
First f/u (Feb 11^th^, 2018)	Same	(Dilated) 20/400, 4/200	17, 18	Conj: Quiet, Cornea: clear, AC: +2 cells/deep Broken posterior synechia OS	No vitritis, ERD mainly inferiorly
2^nd^ f/u (Feb 25^th^, 2018)	Same	(Dilated) 20/400, 4/200	20 OU	Conj: Quiet, Cornea: clear, AC: occasional cells/deep Broken posterior synechia OS	No vitritis, ERD mainly inferiorly with RPE changes
3^rd^ f/u (Mar 18^th^, 2018)	Same	(Dilated) 20/400, 4/200	22 OU	Conj: Quiet, Cornea: clear, AC: occasional cells/deep Broken posterior synechia OS	No vitritis, ERD mainly inferiorly with RPE changes
4^th^ f/u (Apr 1^st^, 2018)	Same	(Dilated) 20/400, CF	14 OU	Conj: Quiet, Cornea: clear, AC: occasional cells/deep Broken posterior synechia OS	No vitritis, ERD mainly inferiorly with RPE changes
5^th^ f/u (Apr 24^th^, 2018)	Same	(Dilated) 20/200, 20/60	13, 14	Conj: Quiet, Cornea: clear, AC: occasional cells/deep Broken posterior synechia OS	No vitritis, flat retina with RPE changes
6^th^ f/u (May 29^th^, 2018)	Same	(PH) 20/30, 20/50	13, 14	Conj: Quiet, Cornea: clear, AC: +1 cells/deep OD, and AC: +2 cells/deep Broken posterior synechia OS	No vitritis, flat retina with RPE changes
7^th^ f/u (Aug 28^th^, 2018)	Same	(PH) 20/50, 20/40	13, 14	Conj: Quiet, Cornea: clear, AC: no cells/deep OD, and AC: occasional cells/deep Broken posterior synechia OS	No vitritis, flat retina with RPE changes
8^th^ f/u (Dec 2^nd^, 2018)	Same	(PH) 20/60, 20/30	18, 17	Conj: Quiet, Cornea: clear, AC: no cells/deep OD, and AC: occasional cells/deep Broken posterior synechia OS	No vitritis, flat retina with RPE changes
9^th^ f/u (Mar 19^th^, 2019)	Same	20/50 OU (PH) 20/30 OU	21 OU	Conj: Quiet, Cornea: clear, AC: no cells/deep OD, and AC: occasional cells/deep Broken posterior synechia OS	No vitritis, ERD mainly inferiorly with RPE changes
10^th^ f/u (Jul 23^rd^, 2019)	Same	20/50 OU (PH) 20/30 OU	21 OU	Conj: Quiet, Cornea: clear, AC: no cells/deep OD, and AC: occasional cells/deep Broken posterior synechia OS	No vitritis, flat retina with RPE changes
11^th^ f/u (Jan 21^st^, 2020)	Same	20/50 OU (PH) 20/40 OU	21 OU	Conj: Quiet, Cornea: clear, AC: no cells/deep OD, and AC: occasional cells/deep Broken posterior synechia OS	No vitritis, flat retina with RPE changes
12^th^ f/u (Aug 25^th^, 2020)	Same	20/40, 20/25	19, 12	Conj: Quiet, Cornea: clear, AC: no cells/deep OD, and AC: occasional cells/deep Broken posterior synechia OS	No vitritis, flat retina with RPE changes, depigmented fundus
13^th^ f/u (Jan 24^th^, 2021)	Same	20/30 OU	15 OU	Conj: Quiet, Cornea: clear, AC: no cells/deep OD, and AC: occasional cells/deep Broken posterior synechia OS	No vitritis, flat retina with RPE changes, depigmented fundus
14^th^ f/u (Jun 08^th^, 2021)	Same	20/30 OU	19, 17.5	Conj: Quiet, Cornea: clear, AC: no cells/deep OD, and AC: occasional cells/deep Broken posterior synechia OS	No vitritis, flat retina with RPE changes, depigmented fundus

**Figure 1:**
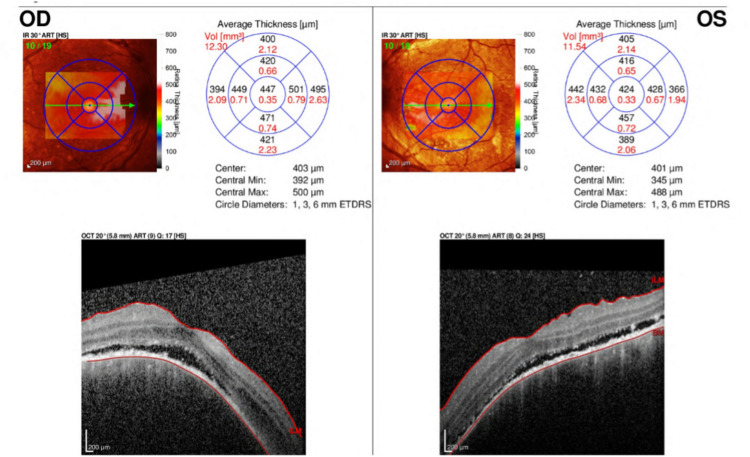
OCT during the first visit. Sub-retinal fluid with shagging of photoreceptor layer. OCT, optical coherence tomography; OD, right eye; OS, left eye

**Figure 2:**
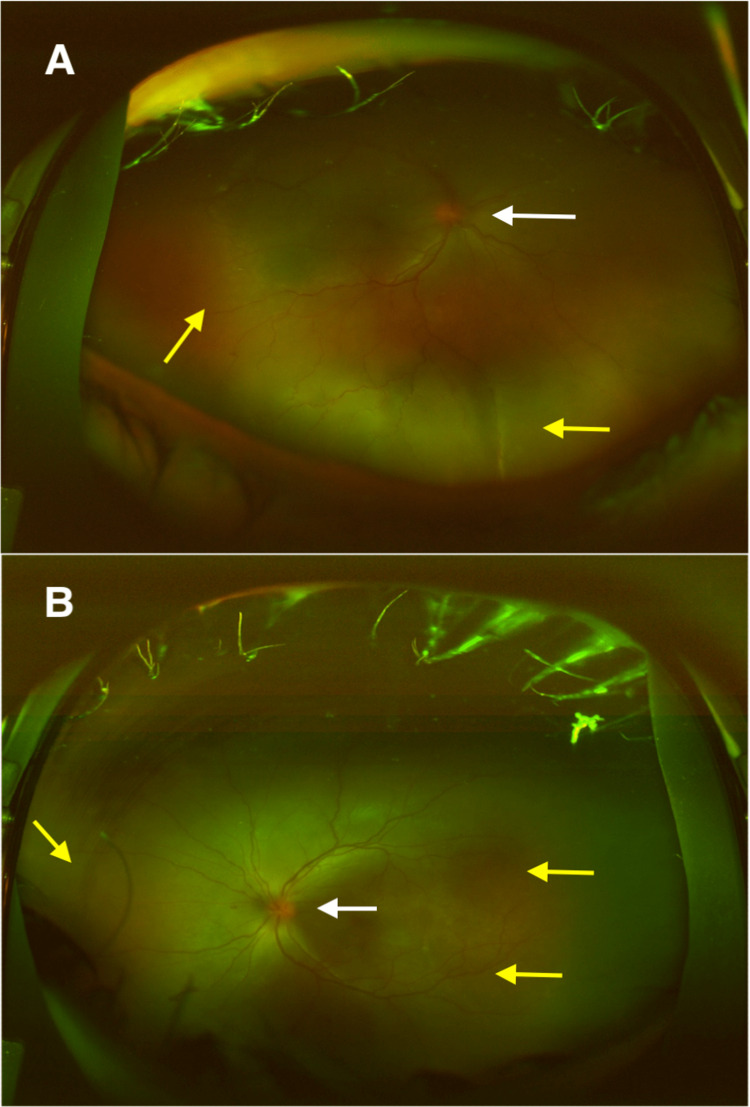
DFE during the first visit. Hyperemic discs with multiple areas of exudative retinal detachments. A: Right eye, B: Left eye, DFE: Dilated fundus examinations, hyperemic discs (white arrows), exudative retinal detachments (yellow arrows)

**Figure 3:**
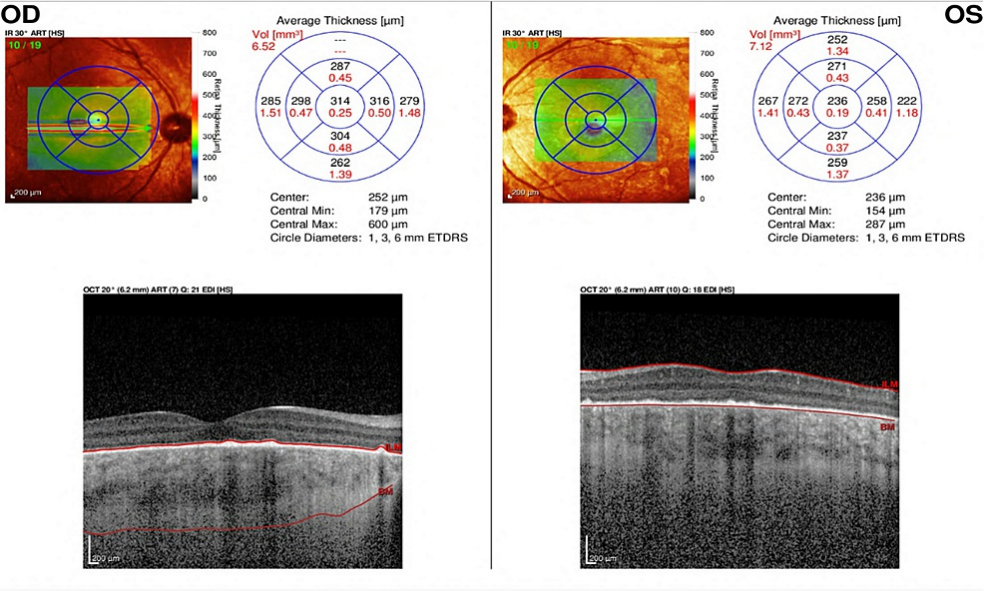
OCT 6 weeks f/u. Complete resolution of sub-retinal fluid with complete loss of photoreceptor layer. OCT, optical coherence tomography; f/u, follow up; OD, right eye; OS, left eye

**Figure 4:**
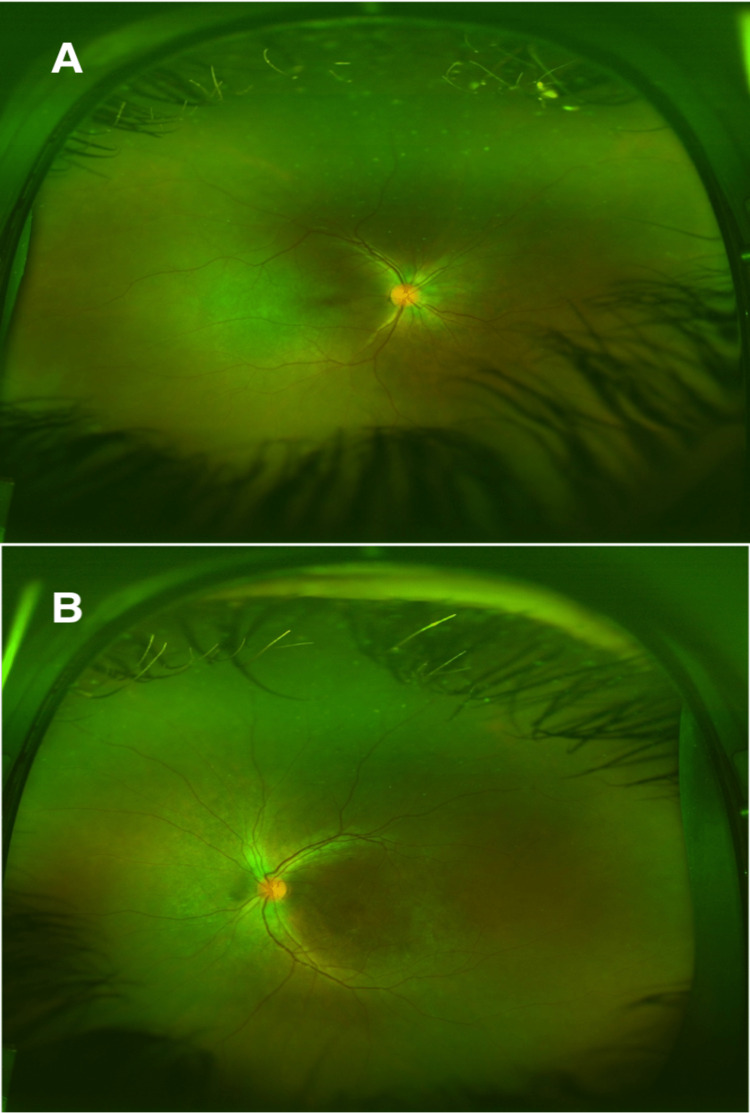
DFE 6 weeks f/u. Normal-looking optic discs without exudative retinal detachments. A: Right eye; B: Left eye; DFE, dilated fundus examinations; f/u, follow up

**Figure 5:**
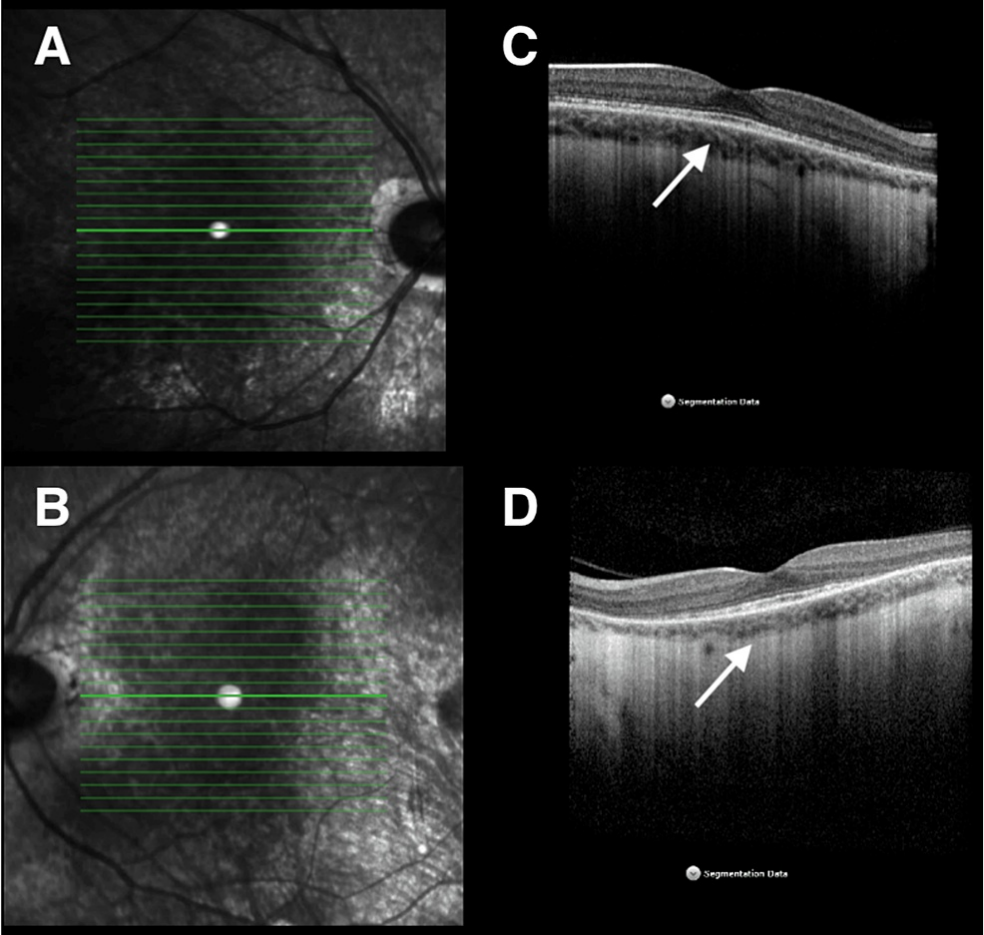
OCT last f/u. Complete resolution of sub-retinal fluid with complete loss of photoreceptor layer. A: Right eye; B: Left eye; C: Right eye; D: Left eye; OCT, optical coherence tomography; f/u, follow up; resolution of sub-retinal fluid (white arrows)

**Figure 6:**
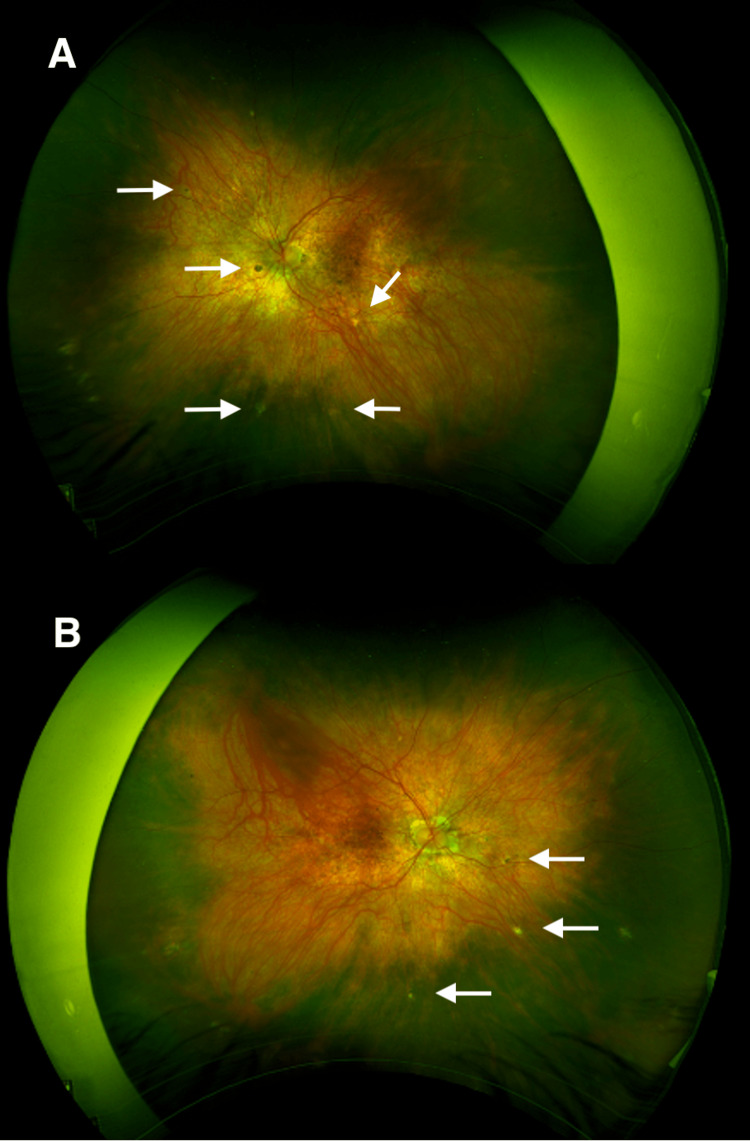
DFE last f/u. Sunset glow fundi, chorioretinal atrophy around optic discs, and Dalen-Fuchs nodules. A, Right eye; B, Left eye; DFE, dilated fundus examinations; f/u, follow up, Dalen-Fuchs nodules (white arrows)

The patient was admitted for uveitis workup, fundus fluorescein angiography, optical coherence tomography, B-scan and to receive the vision-saving IV methylprednisolone after clearance from a pediatrician (Figures 1-6).

Some 500 mg pulse IV methylprednisolone was started 48 h after a negative purified protein derivative (PPD) result and resulted in steroid-induced hyperglycemia as blood glucose level increased from a baseline of 5.4 to 17.6 mmol/L, and a brain imaging showed pituitary gland changes that are compatible with diabetes insipidus. However, the patient’s fasting blood sugar normalized on the same day, and then he received his second dose of 500 mg pulse IV methylprednisolone the following day. On day three of treatment, his fasting blood sugar was stable, so he received his third dose of IV methylprednisolone along with prednisolone acetate every hour along with atropine 1% in both eyes. On the fourth day, he received his fourth and last dose and was then shifted to oral prednisolone 1 mg/kg/day given as a single dose with close observation of his glucose level and was referred to a pediatric endocrinologist and rheumatologist to give clearance for immunosuppressive therapy.

Two weeks later, he was started on adalimumab 0.4 mg/kg/2 weeks and methotrexate 0.4 mg/kg/week along with oral prednisolone 1 mg/kg/day. At his fourth follow-up visit, after two months from treatment initiation, he reported a history of alopecia and hearing problems. At his fifth follow-up visit (one month later), he was already on mycophenolate mofetil 10 mg/kg twice daily and his disease was inactive and oral prednisolone was gradually tapered to 0.1 mg/kg/day. At his sixth follow-up visit (one month later), he was found to have bilateral active anterior uveitis and the plan was to increase prednisolone acetate frequency to four times daily, three times daily, two times daily two weeks each then keep once a day till next visit and to increase mycophenolate mofetil dose to 15 mg/kg twice daily. At his seventh follow-up visit (three months later), the anterior uveitis had subsided, and the plan was to decrease prednisolone acetate to every other day. At his last follow-up visit (three years later), he was on oral prednisolone 0.05 mg/kg/day, mycophenolate mofetil 20 mg/kg twice daily, and prednisolone acetate every other day without any complaints and the ocular inflammation was inactive.

## Discussion

Retinal regeneration is an uncommon outcome that sometimes develops during follow-ups after the resolution of inflammation and uveitis, while it is a well-recognized phenomenon in some other creatures during multiple stages of their development including fishes, birds, and amphibians [3]. Two main causes are responsible for outer segment damage and thinning of the retina in patients with VKH, the first is the inflammation that accompanies the disease and the second is the serous retinal detachment (SRD) [4]. A study evaluated the photoreceptor outer segment volume using a spectral domain OCT and showed a temporary decrease in its volume after the resolution of retinal detachment which then increased in volume with follow-ups. Authors thought that the inflammation resulted in decreased thickness of the outer segment layer and that upon the disappearance of the stimulus, the outer segment enlarged and recovered [5]. In contrast to these findings noticed by an OCTs, the mean cone density increased but did not recover to the control levels following the resolution of SRD even after a 12-month follow-up period of VKH patients using adaptive optics (AO) fundus camera which observed more details than OCT, although visual acuity was well maintained [6].

As a rule, early aggressive treatment with corticosteroids is the mainstay of treatment in the acute uveitis phase as it can reduce the risk of going into a chronic/recurring phase and retinal depigmentation as well as reduce the extraocular manifestations of the disease [1]. Recent studies suggest that combining immunomodulatory therapy with corticosteroids during the acute uveitic phase of VKH disease improves visual outcomes when compared to using corticosteroids alone or with the use of immunosuppressants afterward [7]. The chronic recurrent nature of the pediatric disease, the delay in diagnosis, and the poor tolerance to prolonged systemic corticosteroid therapy make managing VKH syndrome in children difficult and controversial [8-10]. Berker et al., treated their patient with corticosteroid alone, which showed improvement initially. However, the inflammation was exacerbated by any attempt to taper the corticosteroid dose, which they attributed to the patient's delayed diagnosis and treatment [11]. Our patient was started on corticosteroids along with immunosuppressive therapy during the initial active phase, which showed a favorable outcome within three months’ time. Similarly, Soheilian et al. documented 10 VKH syndrome patients who improved or maintained their visual acuity after receiving oral prednisolone and methotrexate [12]. Our patient developed steroid-induced hyperglycemia and diabetes insipidus shortly after corticosteroid use, a drug that has been associated with a wide range of adverse effects that has been increasing in incidence with chronic use [13]. Pituitary gland changes consistent with diabetes insipidus in a VKH patient is an association that was mentioned once in the literature before [14]. Hyperglycemia is a major concern when taking systemic corticosteroids even with short-term use, which is believed to be due to an increase in insulin resistance [15]. A study measured blood glucose levels after pulse dexamethasone for four hours post-drug administration and showed a rise in blood glucose levels within the four-hour period [15].

Central diabetes insipidus (CDI) is a deficiency in vasopressin hormone whereas nephrogenic diabetes insipidus is a resistance to the hormone at the target organ (the kidney) leading to very diluted urine in large amounts [16]. In most case reports found in the literature of patients developing CDI after using corticosteroids, it was believed that the CDI was present before and been masked by glucocorticoid deficiency which then became unmasked by corticosteroid administration leading to a rapid rise in serum sodium and serum osmolarity putting the patient at risk for central demyelination [17]. The mechanism of which involves the effect of glucocorticoid deficiency on impairing free water excretion via arginine vasopressin depended and independent mechanisms [17]. In pituitary lesions and hypocortisolism, doctors should be wary of the unmasking effect of steroid use on the CDI and should have a high index of suspicion when a patient suddenly develops an excessively diluted urine after steroid administration [17].

## Conclusions

Vogt-Koyanagi-Harada is a rare multisystem autoimmune disorder affecting melanocyte-containing tissues. It specifically affects the choroid layer in the eyes. It consists of early acute uveitic manifestations, and late ocular manifestations. Patients with VKH disease might have a good prognosis if treated urgently and aggressively. Retinal photoreceptor layer regeneration can sometimes be observed with follow ups after resolution of inflammation and uveitis.
